# Cellular oxygen consumption, ROS production and ROS defense in two different size-classes of an Amazonian obligate air-breathing fish (*Arapaima gigas*)

**DOI:** 10.1371/journal.pone.0236507

**Published:** 2020-07-30

**Authors:** Bernd Pelster, Chris M. Wood, Derek F. Campos, Adalberto L. Val

**Affiliations:** 1 Institute of Zoology, University of Innsbruck, Innsbruck, Austria; 2 Center for Molecular Biosciences, University Innsbruck, Innsbruck, Austria; 3 Department of Zoology, University of British Columbia, Vancouver, BC, Canada; 4 Department of Biology, McMaster University, Hamilton, ON, Canada; 5 Laboratory of Ecophysiology and Molecular Evolution, Brazilian National Institute for Research of the Amazon, Manaus, Brazil; University of California Davis, UNITED STATES

## Abstract

In air-breathing fish a reduction of gill surface area reduces the danger of losing oxygen taken up in the air-breathing organ (ABO) to hypoxic water, but it also reduces the surface area available for ion exchange, so that ion regulation may at least in part be transferred to other organs, like the kidney or the gut. In the air-breathing *Arapaima gigas*, gill lamellae regress as development proceeds, and starting as a water-breathing embryo *Arapaima* turns into an obligate air-breathing fish with proceeding development, suggesting that ion regulation is shifted away from the gills as the fish grows. In *Arapaima* the kidney projects medially into the ABO and thus, probably a unique situation among fishes, is in close contact to the gas of the ABO. We therefore hypothesized that the kidney would be predestined to adopt an increased importance for ion homeostasis, because the elevated ATP turnover connected to ion transport can easily be met by aerobic metabolism based on the excellent oxygen supply directly from the ABO. We also hypothesized that in gill tissue the reduced ion regulatory activity should result in a reduced metabolic activity. High metabolic activity and exposure to high oxygen tensions are connected to the production of reactive oxygen species (ROS), therefore the tissues exposed to these conditions should have a high ROS defense capacity. Using *in vitro* studies, we assessed metabolic activity and ROS production of gill, kidney and ABO tissue, and determined the activity of ROS degrading enzymes in small (~ 5g, 2–3 weeks old) and larger (~ 670 g, 3–4 months old) *A*. *gigas*. Comparing the three tissues revealed that kidney tissue oxygen uptake by far exceeded the uptake measured in gill tissue or ABO. ROS production was particularly high in gill tissue, and all three tissues had a high capacity to degrade ROS. Gill tissue was characterized by high activities of enzymes involved in the glutathione pathway to degrade ROS. By contrast, the tissues of the ABO and in particular the kidney were characterized by high catalase activities, revealing different, tissue-specific strategies in ROS defense in this species. Overall the differences in the activity of cells taken from small and larger fish were not as pronounced as expected, while at the tissue level the metabolic activity of kidney cells by far exceeded the activity of ABO and gill cells.

## Introduction

Air-breathing fish are often characterized by a significant reduction in gill surface area in order to avoid loss of oxygen taken up in the air-breathing organ (ABO) at the site of the gills in hypoxic water [1–4]. Gills are, however, multifunctional organs, not only responsible for gas exchange, but also for ion regulation, nitrogen excretion, and they are involved in water and acid-base homeostasis, for example. Reducing the surface area of the gills, therefore, has implications for many physiological phenomena (for review see [5]). To understand the transition to air-breathing in teleosts, it is important to address the question how ion or nitrogen homeostasis, for example, can be maintained in spite of a reduction in gill surface area.

In the teleost fish *Arapaima gigas* (Arapaimidae), the reduction of gill surface area can be observed with development. After hatching the gill filaments bear typical gill lamellae, and in a 10-g fish, about 4 weeks after hatching, the lamellae are clearly visible [6]. With further development these lamellae disappear, because they become covered by proliferation of epithelial cells, and their internal pillar cell blood channels exhibit atrophy [7]. In a 100-g fish only rudimentary lamellae are present, and in a 1-kg fish the lamellae are completely gone [6, 7]. As a result, starting as a water breathing embryo, with proceeding development *Arapaima* switches to air-breathing, and finally becomes an obligatory air-breathing fish that drowns if access to air is denied. In a recent study we demonstrated that even 5-g fish regularly breathe air and take 63% of their O_2_ from this phase [8]. Fish of about 60 to 70 g have been reported to take up about 76% of O_2_ from the air, while CO_2_ (86%) is primarily excreted to the water [9], and the partitioning is similar in 600–700 g fish [8].

Even more peculiar is the organization of the kidney in this species. At the dorsal side the elongated kidney projects medially into the ABO, and the kidney is covered by a membrane of the ABO [10]. The close proximity between the air space in the ABO and the kidney is a unique situation among vertebrates. This suggests that physiological implications are involved in this structure, and it has been proposed that in *Arapaima* the kidney plays a particularly important role in ion homeostasis [9]. Due to the lack of the loop of Henle, teleost fish cannot concentrate ions in the urine, but by highly efficient ion resorption *Arapaima* may be able to reduce ion loss via urine production. Ion transport, and in particular ion resorption, which in freshwater fish is based on V-ATPase activity and/or sodium-proton exchange (NHE) [11, 12], require energy and therefore are directly or indirectly coupled to ATP production and turnover.

We therefore hypothesized that if the kidney would significantly contribute to ion regulation metabolic activity of kidney tissue would be particularly high. Metabolic activity of gill tissue, in turn, would be reduced due to a reduced contribution to ion homeostasis. Arterial oxygen partial pressure of water-breathing fish typically is much lower than aerial PO_2_ [13, 14]. High oxygen partial pressures are known to stimulate the production of reactive oxygen species (ROS) [15–18]. Accordingly, exposure of the air-breathing fish *Heteropneustes fossilis* to air caused an increase in ROS production [19, 20]. We therefore also hypothesized that in the air-breathing fish *Arapaima* the close proximity to air in the epithelia of the ABO and in kidney tissue would have implications for ROS production and ROS defense capacities. Specifically, we predicted that in *Arapaima*, tissues routinely exposed to air would be characterized by a high ROS defense capacity, achieved by an elevated activity of ROS degrading enzymes and an elevated concentration of the low molecular weight antioxidant glutathione.

## Materials and methods

Experiments were performed at the Instituto Nacional de Pesquisas da Amazônia (INPA) in Manaus, Brazil. All experimental protocols of this study were in compliance with Brazilian national and INPA animal care regulations (protocol number 026/2015).

*Arapaima gigas* were obtained from a commercial fish culture in Manaus, brought to the INPA and kept in outdoor fish tanks, supplied with running INPA well water. The water was continuously aerated. In the tanks, shaded from sun exposure by a lid, the fish had free access to air. Fish were fed with fish pellets, which were reduced to a powder for small fish, daily until the day before the experiment. Water pH was 6.5–7.0, the temperature was 27–30°C. Body mass of small pirarucu (2–3 weeks old) was 4.75 ± 0.07 g, and total length (measured from the snout to the tip of the rounded caudal fin) 10.2 ± 0.1 cm (N = 24). Body mass of the larger *A*. *gigas* (3–4 months old) was 664 ± 38 g, and total length 47.3 ± 0.7 cm (N = 17).

### Tissue preparation

Fish maintained under normoxic conditions were rapidly anesthetized with an overdose of tricaine methanesulfonate (MS222; 0.5 g L^-1^) and euthanized by a sharp blow on the head for the collection of ABO, kidney and gill tissue. Fish were opened ventrally, and the ABO was exposed. Tissue samples of the ABO and the kidney were rapidly dissected, carefully rinsed with fish saline solution, cleaned and blotted dry. Gill tissue was similarly dissected, cleaned and blotted dry. All tissue samples taken for enzyme and metabolite assays were immediately frozen in liquid nitrogen and then stored in a biofreezer at -80°C until analysis. Tissue preparation and freezing was completed within less than eight minutes from euthanization. Due to the small amount of tissue obtained from 5-g fish, tissues from two fish had to be pooled for biochemical analysis and treated as one single independent sample. Tissue samples collected for determination of cellular oxygen uptake and ROS production were rinsed with BIOPS (see below). These tissue samples were not frozen and were transferred into ice-cold relaxing buffer (see below) within less than five minutes from euthanization, for immediate analysis. Due to the small amount of tissue required for these assays, pooling of samples was not necessary.

### Biochemical analysis

For determination of total glutathione (GSSG + GSH) content, tissue extracts of the frozen tissue samples were prepared using 5% metaphosphoric acid (MPA). The frozen tissues were ground to a fine powder and dissolved 1:5 w/v in 5% MPA. Under ice-cooling, the solution was homogenized using a motorized homogenizer and centrifuged at 13000 rpm for 15 min in an Eppendorf centrifuge, rotor model FA-45-30-11, at 4°C. The supernatant was separated and appropriately diluted using assay buffer for total GSSG+GSH determination. Total GSSG+GSH concentration was determined using the OxiSelect Total Glutathione Assay Kit (STA-312, Cell Biolabs Inc., San Diego, USA), following the manufacturer’s instructions.

For measurement of enzyme activities, the frozen tissue samples were homogenized under ice-cooling in 1:5 w/v of ice-cold homogenization buffer (10 mM TRIS/HCl, 0.1 mM disodium EDTA, 150 mM NaCl, pH 7.5 at 25°C). Homogenates were centrifuged at 13000 rpm for 15 min in an Eppendorf centrifuge, rotor model FA-45-30-11 at 4°C and appropriate dilutions of the supernatant were used for the enzyme and protein assays.

Enzyme activities were measured using a SpectraMax 384Plus microplate spectrophotometer (Molecular Devices, Sunnyvale, CA, USA) at 25 ± 0.1°C. Glutathione reductase (GR; EC 1.6.4.2.) and glutathione peroxidase (GPx; EC 1.11.1.9.) activities were measured using the Glutathione Reductase Assay Kit (No 703202; Cayman Chemical Company, Ann Arbor, USA), and the Glutathione Peroxidase Assay Kit (No 703102; Cayman Chemical Company). Catalase (Cat; EC 1.11.1.6.) activity was assayed using the Amplex Red Catalase Assay Kit (A22180; Molecular Probes, Eugene, USA). Superoxide dismutase (SOD; EC 1.15.1.1.) activity was measured as described by [21]. Briefly, superoxide generated from xanthine in the xanthine oxidase reaction causes a reduction of cytochrome c, which is inhibited by the presence of SOD. One unit of SOD activity is defined as the amount of enzyme (per milligram of protein) that inhibits the reduction of cytochrome c observed in the blank without SOD by 50%. Protein concentration in the homogenate was measured with Coomassie Brilliant Blue G-250 [22] using bovine serum albumin as a standard.

### Determination of cellular oxygen uptake and ROS production

The freshly dissected tissues (ABO, kidney and gills) were immersed in an ice-cold relaxing buffer (BIOPS: 2.8 mM CaK_2_EGTA, 7.2 mM K_2_EGTA, 5.8 mM Na_2_ATP, 6.6 mM MgCl_2_·6H_2_O, 20 mM taurine, 20 mM imidazole, 0.5 mM dithiothreitol, 50 mM K-MES, 15 mM Na-phosphocreatine and 50 mM sucrose, pH 7.2 at 4°C). The tissue was teased into fiber blocks using a dissecting microscope and immersed in 1 ml ice-cold BIOPS along with 50 μg/ml saponin according to [23]. After 30 minutes, fibers were washed three times for 10 min in 2 ml of modified mitochondrial respiratory medium (MiRO5: 0.5 mM EGTA, 3 mM MgCl_2_·6H_2_O, 60 mM K-lactobionate, 20 mM taurine, 10 mM KH_2_PO4, 20 mM HEPES, 160 mM sucrose and 1 g/l BSA, essentially free of fatty acid, pH 7.2) [24]. The fibers were blotted dry on filter paper and weighed into bundles for respiration assays in 2 mL of MiR05 at 28°C. The weight of the tissues (in mg) were 8.52 ± 2.80, 7.70 ± 2.80 and 7.84±1.82 for gills, kidney and ABO respectively in small *A*. *gigas*, and 8.14±1.93, 8.97±1.95 and 10.00 ± 3.68 for gills, kidney and ABO, respectively in larger *A*. *gigas*.

**Fig 1:**
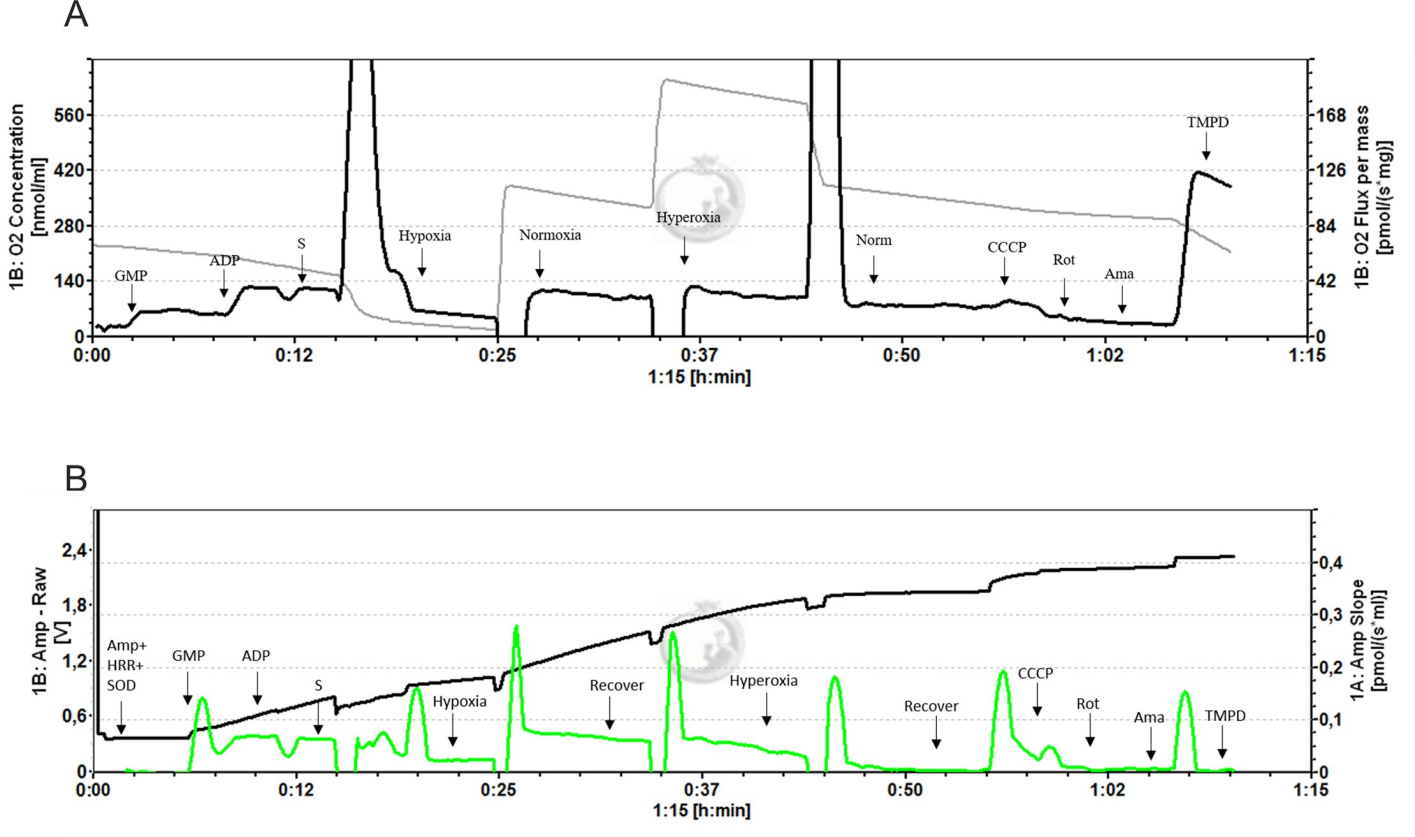
(A) Representative experiment at 28°C on permeabilized kidney cells to measure mitochondrial respiration rate during oxidative phosphorylation. The grey line represents the oxygen concentration in the chamber and the black line is the tissue oxygen consumption. The arrows indicate the steps of the protocol. The addition of GMP (glutamate, malate, and pyruvate) induced the LEAK respiration, addition of ADP induced oxidative phosphorylation (OXPHOS), and the addition of succinate (S), activated complex II. Hypoxia was induced by decreasing oxygen to 2kPa for 10 min, followed by the return to normoxic levels. For hyperoxia, oxygen partial pressure was increased to 50kPa, followed by a return to normoxia. Finally, an uncoupler (CCCP) was added to stimulate maximum phosphorylation. The experiment was terminated by addition of rotenone (Rot) to block the complex I and Antimycin A was added to block complex III in order to measure the background respiration. Complex IV respiration was measured with TMPD and ascorbate as electron donors. (B) Representative experiment at 28°C on permeabilized gill cells to simultaneously measure ROS production. The sample DatLab tracings show cumulative chamber fluorescence (black line, left y-axis) and the rate of chamber fluorescence development (green line, right y-axis) over time during the experimental analyses. Protocol steps as listed above.

Oxygen uptake and ROS production were simultaneously measured in the same cell preparation using the Oroboros Oxygraph and DatLab 2 software (Oroboros Instruments GmbH, Innsbruck, Austria). Under normoxic conditions (PO_2_ = 20 kPa) complex I- (CI) linked respiratory substrates (5 mM malate, 10 mM glutamate and 5 mM pyruvate) were added to measure state II respiration through CI in the absence of ADP (denoted ‘Leak'). By addition of excess ADP (5 mM) oxidative phosphorylation (OXPHOS) was stimulated. Phosphorylating respiration with CI and complex II (CII) substrates were measured by the addition of succinate (20 mM). Hypoxic conditions (PO_2_ = 2 kPa) were established for 10 min by reducing the oxygen supply via bubbling with nitrogen. Post-hypoxia, cells were allowed to recover for 10 min under normoxic conditions, prior to increasing PO_2_ to 50 kPa by addition of pure oxygen for 10 min (hyperoxia). After a recovery period of 10 min in normoxia, CCCP was added to measure uncoupling potential. Complex IV (CIV) activity was recorded by addition of 5 mM ascorbic acid and TMPD (N,N,N',N'-Tetramethyl-p-phenylenediamine dihydrochloride), passing all electrons to complex IV and determining the oxygen uptake following electron flux through complex IV. Standardized calibrations were performed using DatLab 2 software (Oroboros Instruments GmbH, Innsbruck, Austria) following the manufacturer’s instructions. Oxygen consumption was corrected for background respiration determined after addition of rotenone (0.5 μM) and Antimycin A (2.5 μM) to the test medium (Fig 1). ROS emission was measured in parallel with mitochondrial respiration in the same experimental chamber. Superoxide dismutase (SOD; 22.5 U mL^−1^) and horseradish peroxidase (3 U mL^−1^) were added to catalyze the reaction of superoxide produced by the mitochondria and of hydrogen peroxide, respectively, with Ampliflu Red (15 μM) (Oroboros Instruments, Innsbruck, Austria), resulting in the production of the fluorescent product resorufin (detected using an excitation wavelength of 525 nm and ampliometric filter set (AmR); Oroboros Instruments). The resorufin signal was calibrated with additions of exogenous hydrogen peroxide in the MiR05 media before starting the experiment. For that, we added MiR05 to the chamber, then added Ampliflu Red, horseradish peroxidase and SOD. After that, we titrated three times with 0.1 μM H_2_O_2_. Resorufin fluorescence is known to increase over time in the presence of MiRO5 [25]. Values for ROS production therefore were corrected for background fluorescence determined after addition of Antimycin A (Fig 1).

### Statistics

Data have been expressed as mean ± 1 s.e.m. with N giving the number of animals or the number of pooled samples analyzed in each size group. Total GSSG+GSH concentrations are given as μmol g^-1^wwt (wet weight), and enzyme activities as U mg^-1^protein (μmol min^-1^ mg^-1^protein). For statistical analysis of tissue (gill, kidney or ABO) oxygen consumption (MO_2_) three-way repeated measures ANOVA was used, followed by Holm-Sidak multiple comparison procedures. Body mass of the animals (small, larger) was used as parameter (categorical variable) 1, tissue (kidney, gill, ABO) as parameter (categorical variable) 2, and oxygen availability (normoxia (= CI + CII activity in presence of ADP), hypoxia, recovery from hypoxia, hyperoxia and recovery from hyperoxia) were used as parameters (categorical variable) 3. Two-way repeated measures ANOVA followed by Holm-Sidak multiple comparison procedures was used for statistical analysis of ROS production (superoxide + hydrogen peroxide production). Body mass of the animals (small, larger) was used as parameter 1 (categorical variable) and oxygen availability (normoxia (= CI + CII activity in presence of ADP), hypoxia, recovery from hypoxia, hyperoxia and recovery from hyperoxia) were used as parameters (categorical variable) 2. Measured oxygen consumption or ROS production were used as variables (data). Similarly, for enzyme activities and total glutathione concentration, a two-way ANOVA with Holm-Sidak multiple comparison procedures was employed, using body mass as parameter 1, tissue as parameter 2, and activity or concentration as variables. The statistical analysis was performed using the statistical package in SigmaPlot 14.0. Significant differences between values were accepted for p<0.05.

## Results

Oxygen uptake and ROS production have been measured simultaneously in permeabilized cells of the ABO, kidney and gills with the Oroboros Oxygraph as shown in Fig 1.

**Fig 2:**
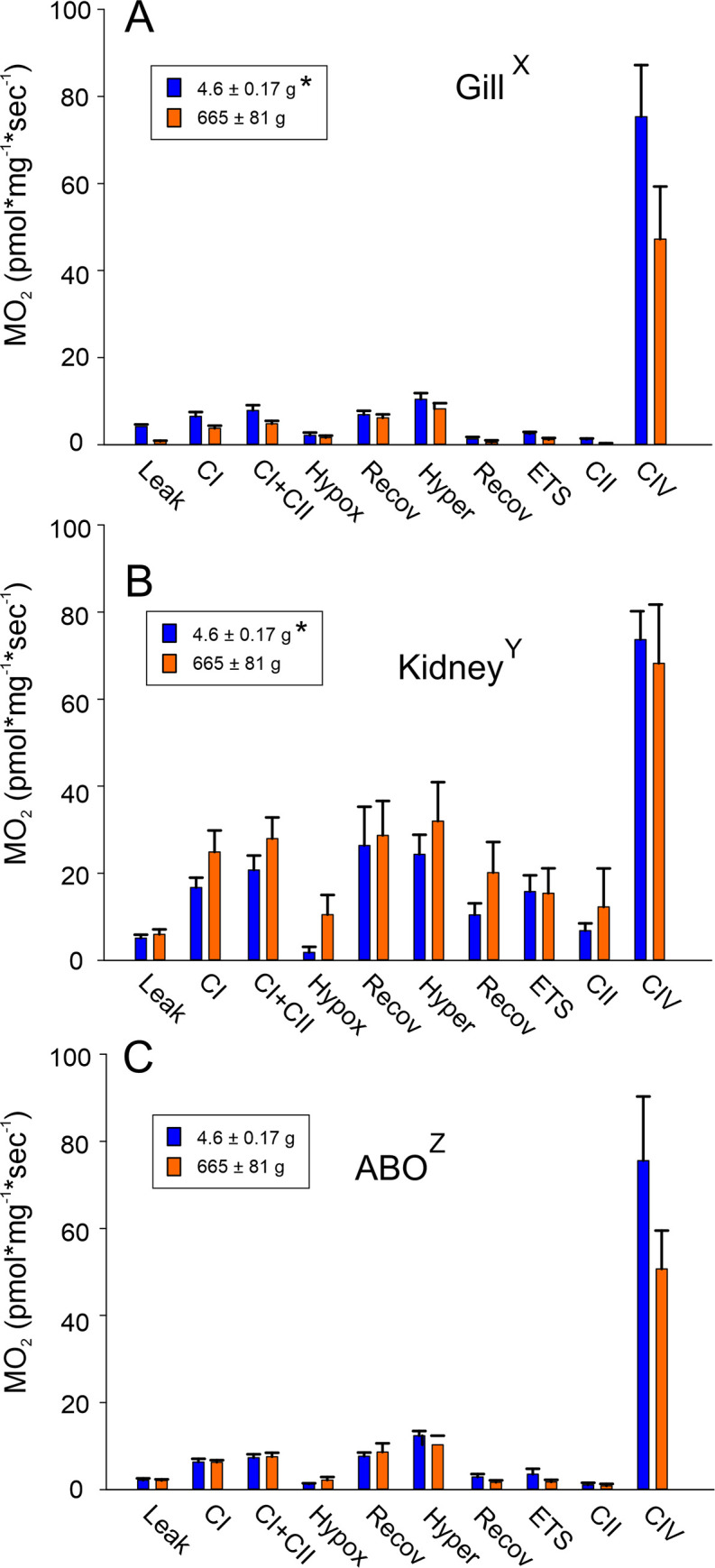
Mitochondrial respiration rates of permeabilized cells of small and larger *Arapaima gigas* gill tissue (A), kidney (B) and ABO (C). Leak respiration was measured in the presence of complex I (CI) substrates (5 mM malate, 10mM glutamate and 5 mM pyruvate), but in the absence of ADP. By addition of excess ADP (5 mM) oxidative phosphorylation was stimulated and CI activity was recorded. CI and complex II (CII) activity was measured by the addition of succinate (20 mM). CI + CII represents the control normoxic condition (20 kPa). Oxygen uptake under hypoxic conditions was measured at a PO_2_ of 2 kPa, while hyperoxic oxygen uptake was recorded at a PO_2_ of 50 kPa. For recovery, PO_2_ was set to normoxia. Oxygen uptake of complex IV (CIV) was measured after addition of 5 mM ascorbic acid and TMPD. * indicates significant overall differences between small and larger fish; capital letters X, Y, Z indicate significant overall differences among tissues; (N = 6; p<0.05).

No difference was detected in oxygen uptake of ABO cells of small and larger fish (Fig 2C). However, overall oxygen uptake was slightly but significantly higher in gill cells of small *A*. *gigas*, while in kidney cells it was significantly higher in the larger fish (Fig 2A and 2B). Significant differences were also observed among tissues. Metabolic activity of kidney cells was approximately 2.6-times higher than the activity of either ABO cells or gill cells in small *A*. *gigas*, and in larger fish this difference was even more pronounced. Hypoxia in all three tissues caused a significant reduction in oxygen uptake. In gill, kidney, and ABO cells of larger fish, oxygen uptake was reduced by more than 60%, in gill and ABO of small fish it was even reduced by more than 70% of the value recorded for CI + CII in the presence of ADP, and in kidney cells to less than 10%. During subsequent normoxic recovery, oxygen uptake returned to the level recorded prior to hypoxia, reaching between 88% and 130% of the value recorded for CI + CII in the presence of ADP. None of these values was significantly different from the oxygen consumption measured prior to the hypoxic bout. In all three cell types, hyperoxia had no significant effect on the rate of oxygen uptake, but during recovery from hyperoxia, in all tissues oxygen uptake initially was reduced as compared to the normoxic values recorded for CI + CII in the presence of ADP (Fig 2A–2C), and in gill and ABO cells this decrease was especially pronounced. Oxygen uptake based on the activity of complex IV was determined at the end of the measurements by addition of TMPD, and in all tissues by far exceeded oxygen consumption rates recorded before. S1 Table lists statistically significant differences in the oxygen uptake recorded during the different steps in the protocol, based on three-way ANOVA.

To confirm the quality of the permeabilized cell preparations, we calculated the respiratory control ratio (RCR = CI/Leak) for all tissues and both fish sizes analyzed, and they showed a high RCR ranging from 3.5 to 6; only in gill cells of small fish a lower value of about 2 was determined (S1 Fig).

**Fig 3:**
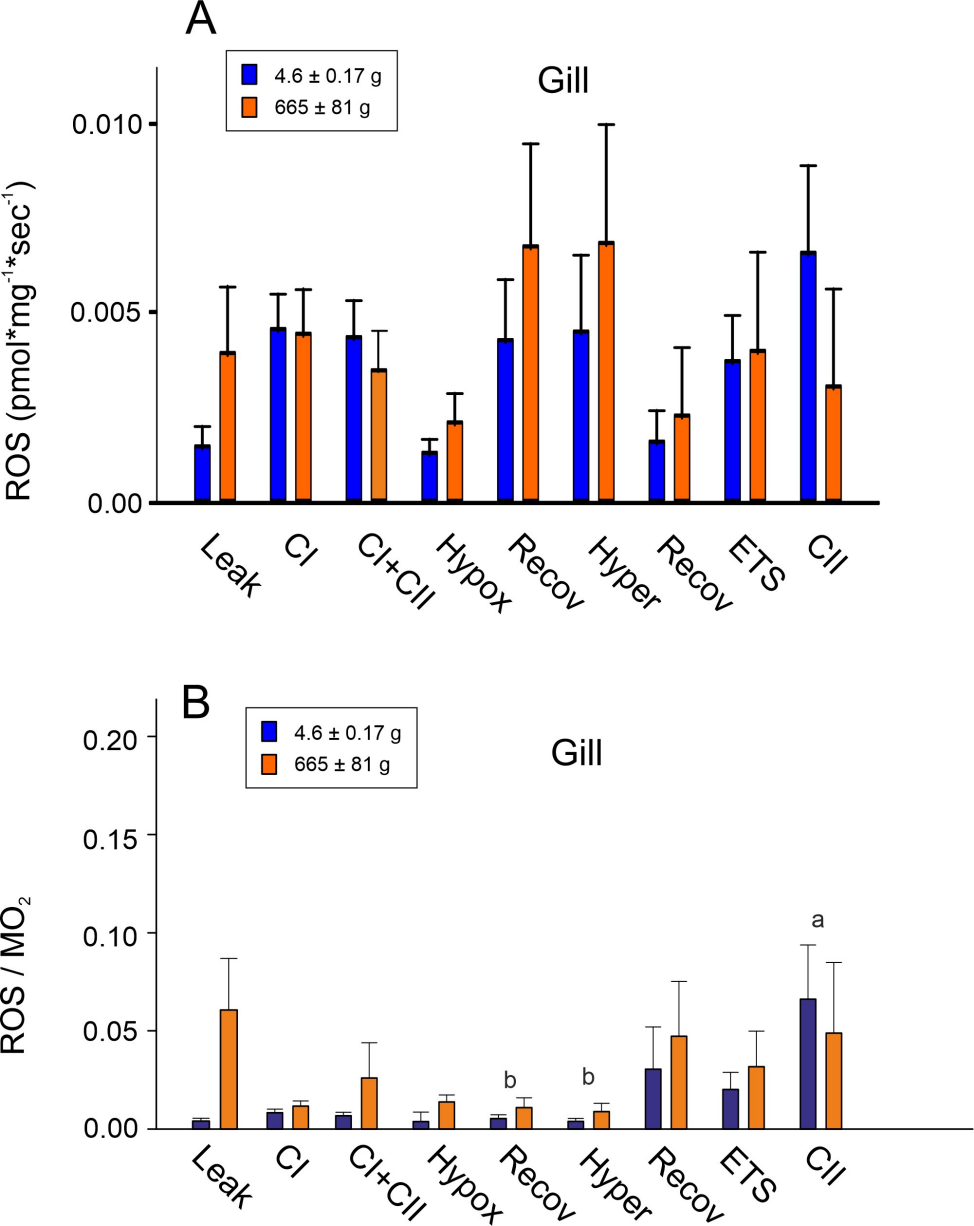
(A) Mitochondrial ROS production of permeabilized gill cells of small and larger *Arapaima gigas*. Measurement conditions were as outlined in Fig 1; (N = 6; p<0.05). (B) Ratio of ROS production related to oxygen consumption (ROS/MO_2_) in small and larger *A*. *gigas* gill tissue. Measurement conditions were as outlined in Fig 1. Different small letters indicate differences among treatments; (N = 6; p<0.05).

ROS production of kidney and ABO cells of small and larger *A*. *gigas* corrected for background fluorescence, resulted in values close to zero, so that ROS production for these two tissues could not be reliably determined. For gill cells, however, cellular ROS production could be determined for small as well as for larger fish (Fig 3A). In gill cells under hypoxic conditions ROS production was lower than ROS production recorded prior to hypoxia under normoxic conditions for the activity of CI + CII in the presence of sufficient ADP (Fig 3A). During normoxic recovery from hypoxia, ROS production returned to previous levels. Hyperoxia did not increase ROS production (Fig 3A).

Oxygen consumption and ROS production were simultaneously measured in the same chamber therefore we could assess a possible connection between the amount of oxygen consumed and the rate of ROS produced by plotting the ratio of ROS production to the rate of oxygen consumption (ROS/MO_2_). There was no difference between larger and small fish (Fig 3B). The ratio was not significantly affected by hyperoxia or by hypoxia; the decrease in oxygen consumption under hypoxic conditions resulted in a proportional decrease in ROS production.

**Fig 4:**
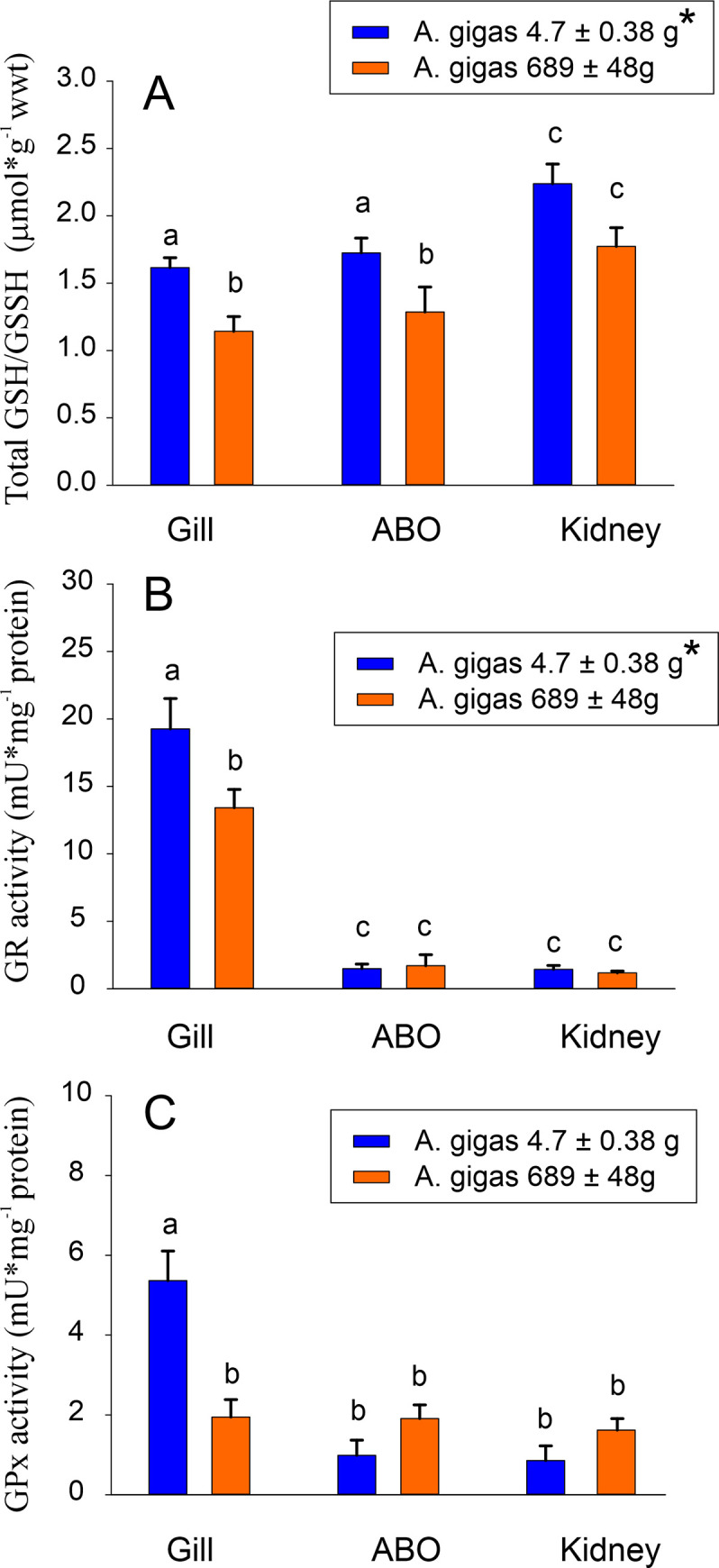
(A) Total GSSG/GSH concentration in small and larger *Arapaima gigas* gill tissue, kidney and ABO. Glutathione reductase (B) and glutathione peroxidase (C) activity in small and larger fish gill tissue, kidney and ABO. * denotes significant overall differences between small and larger fish; different small letters denote significant differences among small and larger fish at the tissue level (N = 6; p<0.05).

To assess the capacity of the three tissues to degrade reactive oxygen species, total glutathione concentration was determined (Fig 4A). In small fish, the total GSSG+GSH concentrations in gill and ABO tissue were significantly elevated compared to larger fish, resulting in an overall significantly higher total glutathione concentration in small *A*. *gigas*. At the tissue level, kidney tissue of both larger and small fish had a significantly higher glutathione concentration than gill tissue and ABO.

**Fig 5:**
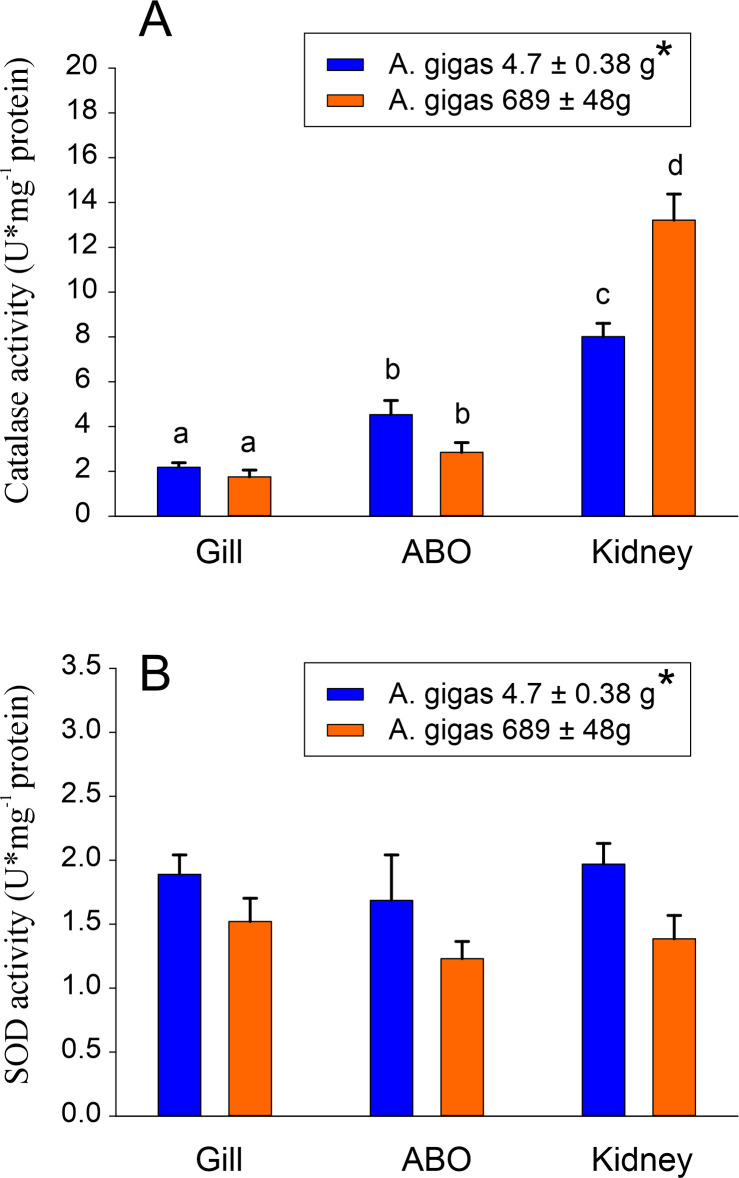
Catalase (A) and SOD (B) activity in small and larger *Arapaima gigas* gill tissue, kidney and ABO. * denotes significant overall differences between small and larger fish; different small letters denote significant differences between small and larger fish at the tissue level (N = 6; p<0.05).

Comparing larger and small *A*. *gigas*, glutathione reductase activity was significantly higher in small *A*. *gigas* (Fig 4B). At the tissue level, gill tissue overall showed significantly higher GR activities than the other two tissues. GPx activity was also elevated in gills of small *A*. *gigas* (Fig 4C). Catalase activity was highest in kidney tissue, and the highest activity was detected in the kidney of larger fish (Fig 5A). Catalase activity in the ABO was elevated overall as compared to gill tissue, but there was no difference between larger and small fish. Overall SOD activity was significantly higher in small fish, but there were no significant differences in SOD activity among the three tissues in small or larger fish (Fig 5B).

## Discussion

### Oxygen consumption

While oxygen uptake of small and larger *A*. *gigas* clearly scaled with body mass [8, 9], oxygen uptake rates of the isolated and permeabilized ABO cells were not different between small and larger fish. Comparing the tissues, however, revealed a very high oxygen consumption of kidney tissue in comparison to gill tissue and ABO (more than 2.6-fold higher). The high metabolic activity was consistent with high Na^+^/K^+^-ATPase activities previously reported for this organ. Na^+^/K^+^-ATPase activity of air-breathing *A*. *gigas* kidney was significantly higher than in the closely related water breather *Osteoglossum bicirrhosum* [26], and the activity detected in kidney tissue also by far exceeded the activity detected in gill cells of *A*. *gigas*, especially in smaller fish [9]. Our own measurements confirmed that Na^+^/K^+^-ATPase activity in the kidney is about ten times higher than in gill or ABO tissue, and V-ATPase activity in kidney tissue also by far exceeds the activity recorded in gill or ABO [8] (Wood, Pelster, Braz-Mota, and Val, unpubl.data). In a previous study it has been proposed that the kidney of *A*. *gigas* plays a very important role in ion regulation by effectively removing ions from the urine [9]. In freshwater fish ion uptake against a concentration gradient ultimately is driven by ATPase activity and therefore requires a great amount of energy. The high rate of oxygen consumption by permeabilized kidney cells measured in our study clearly supports the idea that the kidney in *A*. *gigas* plays an important role in ion homeostasis. Metabolism of the kidney likely benefits from the peculiar arrangement of the organ and the intimate contact to air, and thus to a rich oxygen supply allowing aerobic ATP production and high ATP turnover rates required for effective ion transport.

In contrast to the kidney, the oxygen uptake of gill tissue was low. Indeed, it was in the same range as oxygen uptake of ABO cells, which are involved in gas transfer, but cannot in any way contribute to ion exchange with the environment. This suggests that, compared to water breathing teleosts, the gills of *A*. *gigas* are of reduced importance for ion uptake and ion homeostasis. This is in line with our measurements of Na^+^/K^+^-ATPase activity and also of V-ATPase activity in gill tissue and ABO, which revealed that the activity of both ATPases compared to activities recorded in water breathing fish or even the air-breathing *Hoplerythrinus unitaeniatus* are particularly low, pointing to a low ion exchange capacity of *A*. *gigas* gills, in small as well as in larger fish [8, 27]. In accordance, the gills' oxygen uptake of *A*. *gigas* is lower compared to the water-breathing brown trout (*Salmo trutta*) [28]. Interestingly, the RCR of gills of larger fish is greater than that of small fish. The gill lamellae regress as development proceeds and this decreases the relative surface area available for gas exchange, because they become covered by proliferation of epithelial cells. Therefore, as fish grow, the gill oxygen supply to the gill tissue itself may become limited, and to maintain the physiological function at lower oxygen tension, the gills' respiration increases the phosphorylation efficiency, which means that they produce more ATP per oxygen consumed. This is in line with hypoxia exposure in the Pacific oyster, *Crassostrea gigas*, which increase RCR and ADP/O when facing hypoxia exposure [29]. Furthermore, the gills from small fish showed higher respiration rate and leak respiration. Previous work has shown that during cellular proliferation there is an increase of the activity of uncoupling proteins (UCPs), which induces the proton leak and constrains the oxidative phosphorylation, but limits oxidative cell injury by decreasing ROS production [30].

In all three tissues of small and larger *A*. *gigas*, hypoxia caused a significant decrease in oxygen uptake. At a PO_2_ of 2 kPa, oxygen uptake was reduced generally by more than 60%, and in tissues of small fish an even greater 72% reduction was observed. This reduction may, in part, have been compensated by anaerobic metabolism, but due to the more than 10-fold difference in ATP production between aerobic and anaerobic metabolism hypoxia most likely also resulted in a metabolic depression [31–33]. During subsequent recovery, cells rapidly returned to normoxic oxygen consumption levels. Therefore, our results provide no indication for a significant elevation of oxygen consumption during recovery from hypoxia, which would have been indicative of an oxygen debt encountered during the hypoxic period. This observation suggests that the cells reduced their ATP turn-over during hypoxia and obviously tolerated and easily compensated the short bouts of hypoxia.

Hyperoxia did not significantly enhance oxygen uptake in the three tissues as compared to previous normoxic values. MO_2_ values measured as CI + CII respiration in the presence of ADP, during recovery from hypoxia and during hyperoxia were not significantly different. In consequence, surplus oxygen availability did not increase electron flux through the respiratory chain. This value is, however, far below the oxygen consumption recorded by maximal stimulation of complex CIV, determined in the presence of TMPD. While in kidney cells during recovery from hyperoxia oxygen uptake almost returned to previous normoxic levels, in gill and ABO a significant reduction in oxygen uptake following hyperoxia was observed, suggesting that in these two tissues hyperoxia was not as well tolerated by the cells as hypoxia. Especially in gill cells, oxygen uptake was extremely low after the hyperoxic bout. Subsequent normoxic maximal stimulation of complex CIV, however, again resulted in very high rates of oxygen consumption. Therefore, our findings suggest that hyperoxia influences tissue metabolism by decreasing proton pump and electron transfer activity of the upstream complexes, such as CI and CII.

Mitochondrial proton leak can make a significant contribution to standard metabolic rate as for example shown for rat [34, 35], but on the positive side it can help to prevent the production of ROS [35]. Gill tissue of small *A*. *gigas* was characterized by a high rate of proton leak respiration recorded in the absence of ADP. In larger fish it was significantly reduced. Metabolic efficiency is plastic, and especially shortly after hatching development may play a role. The switch from the water-breathing hatchling to breathing air with a much better oxygen supply occurs within less than two weeks, as we have found that even 4–6 g fish take up 63% of their oxygen from the air [8]. In turn, this switchover is likely to involve significant changes in the electron flux through the respiratory chain. It therefore could be that the coupling of the complexes transporting electrons from complex I to complex IV in the respiratory chain initially is not really tight and is improved with development.

### ROS production and ROS defense

Exposure of tissues to higher levels of oxygen results in the generation of reactive oxygen species (ROS) [15–18]. Previous studies comparing ROS production in various tissues by recording the resulting damage (e.g. lipid peroxidation or protein carbonylation) in fish suggested that liver and kidney are much more prone to ROS production than muscle cells, for example [36–38]. Surprisingly our results revealed that ROS production in permeabilized kidney and ABO cells was very low and could not reliably be measured. In gill cells, however, ROS production was much higher and could be recorded. In gill cells of larger fish, the ratio of ROS/MO_2_ was always higher than in cells of small fish. Gills are typically exposed to water, and therefore experience lower PO_2_ values than the air-breathing organs. Due to the low diffusibility of oxygen in water, PO_2_ at the water surface may be equilibrated with air, but with increasing distance from the water surface, PO_2_ often is reduced, as in the Amazon where hypoxic conditions are frequently encountered. In the air-breathing *A*. *gigas*, however, gills or at least part of the gills most likely will be exposed to air during a breathing cycle. Air is sucked in through the mouth [39] and must pass through the gill chamber to enter the esophagus and the ABO. At the end of an air-breath, gas bubbles are always released through the opercula, and these gas bubbles therefore must also pass the gills. With the reduction in gill lamellae, passage of a gas bubble will not create any problem with a collapse of lamellar structures, but the high oxygen tensions may of course stimulate ROS production.

To avoid damage as a result of ROS accumulation, tissues protect themselves by accumulation of low molecular weight antioxidants such as ascorbate or glutathione, and by the expression of enzymes that can rapidly degrade ROS [15, 16, 40–42]. Previous studies revealed that using the swimbladder as a respiratory organ coincides with an elevation in the ROS defense capacity, as shown by a comparison of the facultative air-breathing erythrinid fish *Hoplerythrinus unitaeniatus* (jeju) with the water breathing erythrinid *Hoplias malabaricus* (traira) [43, 44]. In *A*. *gigas* ABO tissue, but also in kidney tissue, we therefore expected an elevated ROS defense capacity. Like the ABO tissue itself, the kidney, which runs medially through the ABO, is in close contact to air in *A*. *gigas*, and in addition, its oxygen consumption is particularly high. Mitochondria are the main source of ROS [41, 45, 46], and the elevated metabolic activity observed in kidney tissue may therefore contribute to ROS production. Our results show that gill tissue, kidney and ABO of *A*. *gigas* have a high capacity for ROS degradation, but the tissues use different strategies to break down ROS. In gill tissue the concentration of total GSH+GSSG was in the range of 1–2 μmol g^-1^wwt, much higher than in swimbladder tissue of the jeju or traira [43]. In addition, GR activity was almost ten-times higher in gill tissue relative to kidney and ABO, and in gills of small fish GPx activity was also elevated. A glutathione based ROS defense has previously been detected in the air-breathing organ of the jeju [43, 44]. In the jeju swimbladder, total GSH+GSSG concentration as well as GR and GPx activities were elevated as compared to the closely related but water breathing traira. In *A*. *gigas* total GSH+GSSG concentration and GR activity of gill cells even by far exceeded the values determined for jeju swimbladder, revealing a very high glutathione-based ROS defense capacity.

In the ABO and kidney, ROS defense capacity also was remarkably high, but appeared to be mainly based on catalase activity. In the ABO, catalase activity was twice as high as in gill cells, and by far the highest activity was recorded in the kidney, especially in larger fish. In the kidney of larger fish, catalase activity even by far exceeded the activity recorded in the swimbladder of the jeju [43].

In the swimbladder of the European silver eel *Anguilla anguilla*, which is used as buoyancy organ in this species, SOD activity has been shown to be important for the degradation of ROS [47]. In *A*. *gigas*, SOD exhibited similar activity levels in all tissues analyzed and was significantly lower than in the swimbladder of the jeju [43]. Different fish species and fish tissues obviously use the whole array of available strategies to defend against ROS. The consistently high ROS defense capacity detected in the swimbladder of the jeju and in the ABO of *A*. *gigas* indicates that in fish, air-breathing evolved in conjunction with an elevated ROS defense capacity in tissues that experience contact with the air.

ROS production in *A*. *gigas* kidney and ABO cells could not reliably be measured, indicating that in these tissues ROS production was very low. Given the high catalase activity detected in these two tissues it appears possible, that immediate enzymatic degradation of ROS also prevented any accumulation of ROS. This would support the conclusion that these two tissues have a very high ROS defense capacity. Noteworthy was the rapid recovery of kidney cells from hyperoxia. This result was unexpected, given the high metabolic activity manifest in the recorded high oxygen consumption rate and the proximity to the gas space in the ABO. The data thus demonstrate that *A*. *gigas* kidney cells are characterized by tight coupling of the electron transport chain, which allows high metabolic flux with low electron leakage and low ROS production, combined with a high capacity to defend against ROS.

The question whether ROS production increases or decreases under hypoxia has been frequently addressed with conflicting results. Several studies report a paradoxical ROS increase under hypoxia [48–51], while others, including studies on fish, report a decrease in ROS production [52, 53]. Hypoxia acclimation, for example, reduced mitochondrial ROS production in killifish liver cells [54]. Our results show a decreased ROS production in gill tissue under hypoxic conditions *in vitro*, and a return to previous levels on return to normoxia. In gill tissue, the rate of ROS production appeared to be dependent on the rate of oxygen consumption. This was supported by the response to hyperoxic conditions. Hyperoxia did not stimulate ROS production in gill tissue, and MO_2_ remained constant under these conditions. This is also supported by the observation that in kidney and ABO tissue even hyperoxic conditions did not enhance ROS production so that it could be measured. This observation is in line with a previous study, demonstrating that in the air-breathing jeju, hyperoxic exposure hardly affected the ROS defense capacity of swimbladder tissue [44].

The results of our study clearly support the assumption that the kidney of *A*. *gigas* plays an important role in ion homeostasis, while the contribution of the gill appears to the reduced. In spite of the reduced metabolic activity of gills, the branchial tissue shows a high ROS production. Gill, ABO and kidney show a high capacity for ROS degradation, based on either the glutathione-dependent pathway or on catalase activity. Our results also confirm the hypothesis that air-breathing fish are characterized by a high ROS defense capacity in tissues routinely exposed to air.

## Supporting information

S1 Fig The respiratory coupling ratio (RCR = CI/Leak) of different permeabilized tissues from small and larger *A*. *gigas*. The asterisks indicates significant differences (t-test) between small and larger fish within the tissue.
(DOCX)

S1 Table Statistical analysis (three-way ANOVA followed by Holm-Sidak multiple comparison procedure) for oxygen consumption of permeabilized gill, kidney and ABO cells of small and medium sized *A*. *gigas*, presented in Fig 2A–2C. For factor oxygen availability only significant differences are listed.
(DOCX)
