# Supplementary material for: Cellular oxygen consumption, ROS production and ROS defense in two different size-classes of an Amazonian obligate air-breathing fish (Arapaima gigas)
Source: PLoS One. 2020 Jul 30;15(7):e0236507. doi: 10.1371/journal.pone.0236507 (PMC7392269; doi:10.1371/journal.pone.0236507)
Supplement: S1 Fig — The asterisks indicates significant differences (t-test) between small and larger fish within the tissue. (DOCX) [file pone.0236507.s001.docx]

Supplementary Figure 1

The respiratory coupling ratio (RCR = CI/Leak) of different permeabilized tissues from small and larger *A. gigas*. The asterisks indicates significant differences (t-test) between small and larger fish within the tissue.
