# Supplementary material for: Cellular oxygen consumption, ROS production and ROS defense in two different size-classes of an Amazonian obligate air-breathing fish (Arapaima gigas)
Source: PLoS One. 2020 Jul 30;15(7):e0236507. doi: 10.1371/journal.pone.0236507 (PMC7392269; doi:10.1371/journal.pone.0236507)
Supplement: S1 Table — For factor oxygen availability only significant differences are listed. (DOCX) [file pone.0236507.s002.docx]

**Supplementary Table 1**

Statistical analysis (Three-way ANOVA followed by Holm-Sidak multiple comparison procedure) for oxygen consumption of permeabilized gill, kidney and ABO cells of small and medium sized *A. gigas*, presented in Figure 2A, B, C. For factor oxygen availability only significant differences are listed.

**Comparison Diff of Means t P P<0,050**

small vs. medium sized 0,929 0,760 0,448 No

Comparisons for factor: **tissue**

**Comparison Diff of Means t P P<0,050**

kidney vs. gill 12,736 8,505 <0,001 Yes

kidney vs. ABO 11,770 7,859 <0,001 Yes

ABO vs. gill 0,966 0,645 0,519 No

Comparisons for factor tissue: **within small**

**Comparison Diff of Means t P P<0,050**

kidney vs. gill 8,338 3,937 <0,001 Yes

kidney vs. ABO 8,156 3,851 <0,001 Yes

ABO vs. gill 0,182 0,0858 0,932 No

Comparisons for factor tissue: **within medium sized**

**Comparison Diff of Means t P P<0,050**

kidney vs. gill 17,134 8,090 <0,001 Yes

kidney vs. ABO 15,383 7,264 <0,001 Yes

ABO vs. gill 1,751 0,827 0,409 No

Comparisons for factor oxygen availability **within kidney**

**Comparison Diff of Means t P P<0,050**

CIV vs. hypox 1,281 9,281 <0,001 Yes

CIV vs. leak 1,126 8,840 <0,001 Yes

CIV vs. CII 1,066 8,171 <0,001 Yes

hyperox vs. hypox 0,842 6,099 <0,001 Yes

hypo-rec vs. hypox 0,814 5,896 <0,001 Yes

CI+CII vs. hypox 0,802 5,810 <0,001 Yes

CIV vs. hyper-rec 0,719 5,512 <0,001 Yes

hyperox vs. leak 0,687 5,390 <0,001 Yes

CI vs. hypox 0,723 5,237 <0,001 Yes

CIV vs. ETS 0,680 5,208 <0,001 Yes

hypo-rec vs. leak 0,658 5,170 <0,001 Yes

CI+CII vs. leak 0,647 5,077 <0,001 Yes

hyperox vs. CII 0,627 4,805 <0,001 Yes

hypo-rec vs. CII 0,599 4,589 <0,001 Yes

CI+CII vs. CII 0,587 4,499 <0,001 Yes

CI vs. leak 0,567 4,455 <0,001 Yes

CIV vs. CI 0,558 4,384 <0,001 Yes

ETS vs. hypox 0,602 4,269 <0,001 Yes

hyper-rec vs. hypox 0,562 3,986 0,002 Yes

CI vs. CII 0,508 3,892 0,003 Yes

CIV vs. CI+CII 0,479 3,762 0,005 Yes

CIV vs. hypo-rec 0,467 3,670 0,007 Yes

CIV vs. hyperox 0,439 3,449 0,015 Yes

ETS vs. leak 0,446 3,419 0,016 Yes

hyper-rec vs. leak 0,406 3,114 0,042 Yes

Comparisons for factor oxygen availability **within ABO**

**Comparison Diff of Means t P P<0,050**

CIV vs. CII 1,998 14,469 <0,001 Yes

CIV vs. hypox 1,605 12,598 <0,001 Yes

CIV vs. ETS 1,489 11,694 <0,001 Yes

CIV vs. leak 1,453 11,408 <0,001 Yes

CIV vs. hyper-rec 1,413 10,823 <0,001 Yes

hyperox vs. CII 1,275 9,236 <0,001 Yes

hypo-rec vs. CII 1,125 8,151 <0,001 Yes

CI+CII vs. CII 1,102 7,982 <0,001 Yes

CIV vs. CI 0,965 7,578 <0,001 Yes

CI vs. CII 1,032 7,477 <0,001 Yes

CIV vs. CI+CII 0,896 7,032 <0,001 Yes

hyperox vs. hypox 0,882 6,926 <0,001 Yes

CIV vs. hypo-rec 0,872 6,848 <0,001 Yes

hyperox vs. ETS 0,767 6,021 <0,001 Yes

hypo-rec vs. hypox 0,732 5,750 <0,001 Yes

hyperox vs. leak 0,731 5,736 <0,001 Yes

CIV vs. hyperox 0,723 5,673 <0,001 Yes

CI+CII vs. hypox 0,709 5,567 <0,001 Yes

hyperox vs. hyper-rec 0,690 5,287 <0,001 Yes

CI vs. hypox 0,639 5,020 <0,001 Yes

hypo-rec vs. ETS 0,617 4,846 <0,001 Yes

CI+CII vs. ETS 0,594 4,662 <0,001 Yes

hypo-rec vs. leak 0,581 4,560 <0,001 Yes

CI+CII vs. leak 0,557 4,376 <0,001 Yes

hyper-rec vs. CII 0,585 4,150 <0,001 Yes

hypo-rec vs. hyper-rec 0,540 4,140 <0,001 Yes

CI vs. ETS 0,524 4,115 <0,001 Yes

CI+CII vs. hyper-rec 0,517 3,961 0,002 Yes

leak vs. CII 0,545 3,944 0,002 Yes

CI vs. leak 0,488 3,830 0,003 Yes

ETS vs. CII 0,508 3,681 0,004 Yes

CI vs. hyper-rec 0,447 3,427 0,010 Yes

Comparisons for factor: **Col 4 within gill**

**Comparison Diff of Means t P P<0,050**

CIV vs. CII 2,070 16,252 <0,001 Yes

CIV vs. hyper-rec 1,970 15,466 <0,001 Yes

CIV vs. ETS 1,584 12,436 <0,001 Yes

CIV vs. leak 1,495 11,736 <0,001 Yes

CIV vs. hypox 1,488 11,405 <0,001 Yes

hyperox vs. CII 1,275 10,012 <0,001 Yes

hyperox vs. hyper-rec 1,175 9,225 <0,001 Yes

hypo-rec vs. CII 1,134 8,902 <0,001 Yes

CI+CII vs. CII 1,102 8,649 <0,001 Yes

CIV vs. CI 1,059 8,314 <0,001 Yes

hypo-rec vs. hyper-rec 1,034 8,116 <0,001 Yes

CI vs. CII 1,011 7,938 <0,001 Yes

CI+CII vs. hyper-rec 1,002 7,863 <0,001 Yes

CIV vs. CI+CII 0,968 7,603 <0,001 Yes

CIV vs. hypo-rec 0,936 7,350 <0,001 Yes

CI vs. hyper-rec 0,911 7,152 <0,001 Yes

CIV vs. hyperox 0,795 6,241 <0,001 Yes

hyperox vs. ETS 0,789 6,195 <0,001 Yes

hyperox vs. leak 0,700 5,495 <0,001 Yes

hyperox vs. hypox 0,694 5,314 <0,001 Yes

hypo-rec vs. ETS 0,648 5,086 <0,001 Yes

CI+CII vs. ETS 0,616 4,833 <0,001 Yes

leak vs. CII 0,575 4,517 <0,001 Yes

hypox vs. CII 0,582 4,456 <0,001 Yes

hypo-rec vs. leak 0,559 4,386 <0,001 Yes

hypo-rec vs. hypox 0,552 4,232 <0,001 Yes

CI+CII vs. leak 0,526 4,133 <0,001 Yes

CI vs. ETS 0,525 4,122 <0,001 Yes

CI+CII vs. hypox 0,520 3,985 0,001 Yes

ETS vs. CII 0,486 3,816 0,003 Yes

leak vs. hyper-rec 0,475 3,730 0,003 Yes

hypox vs. hyper-rec 0,481 3,689 0,004 Yes

CI vs. leak 0,436 3,422 0,009 Yes

CI vs. hypox 0,430 3,291 0,013 Yes

ETS vs. hyper-rec 0,386 3,030 0,029 Yes
